# Strengthened human resources in health logistics in Nepal

**DOI:** 10.1186/2052-3211-7-S1-P13

**Published:** 2014-12-17

**Authors:** Heem S Shakya, Aneeva Shakya, Umesh K Gupta, Mingmar G Sherpa

**Affiliations:** 1 UNFPA, Jakarta, Indonesia; 2 Oxford University Clinical Research Unit, Kathmandu, Nepal; 3 Population Services International (PSI), Kathmandu, Nepal; 4 Department of Health Services, Ministry of Health and Population, Kathmandu, Nepal

## Background

Prior to 1993, Nepal had a vertical health logistics system. Logistics was not a government priority. No logistics curricula had been developed, no staff had been trained, and no logistics information systems existed at any level. After the establishment of the Logistics Management Division in 1993, the lack of trained manpower in logistics was realized. With support of USAID funded projects (implemented by John Snow Incorporated through Family Planning Logistics Management (FPLM), Nepal Family Health Program, and DELIVER), logistics training was institutionalized within the National Health Training Centre of Ministry of Health and Population.

## Method

With support from USAID, the National Health Training Centre and Logistics Management Division have worked to institutionalize logistics training. Trainers were trained and Regional Health Training centres have been conducting logistics training. Logistics training is included in National Health Training Centre’s annual work plan and approved by the National Planning commission. Logistics practices have been incorporated in the pre-service and in-service curricula and health logistics training has also been incorporated in the training management guideline of the National Health Training Centre.

## Results

Logistics practices have been incorporated in the pre-service and in-service curricula. Technical assistance is being provided to establish or maintain training within a national training system and efforts are being made to build the capacity of government healthcare providers to manage training events. Ten standardized Health Logistics Training packages have been institutionalized into the National Health Training Centre system to train human resource needed for the logistics management of the country and computer based self-paced training (CD-ROM), has been developed.

## Discussion

The Ministry of Health has recognized the importance of the not only logistics management but also the need for quality training. The Ministry of Health has initiated and is continuing provision of logistics training from its own financial resources ensuring sustainability of the program to some extent. From 1993 to 2013, a total of 27,734 government personnel have been trained in the health logistics trainings. Through the training important logistics interventions like Pull System of Health Commodities and web-based LMIS were successfully implemented in all 75 districts of the country.

## Lessons learned

Frequent turnover of trained storekeepers and a lack of effective supervision after training remain concerns. The misconception among health workers that training will solve all performance problems hinders their ability to analyse gaps and subsequently address them; and overall governance and accountability of the Government are continued issues.

